# Renegotiating *situativity*: transformations of local herbal knowledge in a Western Alpine valley during the past 40 years

**DOI:** 10.1186/s13002-020-00402-3

**Published:** 2020-10-01

**Authors:** Michele F. Fontefrancesco, Andrea Pieroni

**Affiliations:** 1 University of Gastronomic Sciences, Piazza Vittorio Emanuele 9, 12060 Bra, Pollenzo Italy; 2 Department of Medical Analysis, Tishk International University, Qazi Muhammad, Erbil, Kurdistan 44001 Iraq

**Keywords:** Ethnobotany, Alps, LEK, Social change, Mountain development

## Abstract

**Background:**

Mountain environments are fragile socio-ecological systems and the conservation of their biological and cultural diversities— seen as co-evolving, strongly intertwined entities—represents a crucial issue for fostering their sustainability. Very few ethnobiological studies have assessed in the mountainous regions of Europe how local botanical knowledge, which represents a vital portion of the local environmental knowledge (LEK), changes over time, although this may be quintessential for a better understanding of the factors influencing how knowledge and practices are shaped, eroded, or even re-created.

**Methods:**

In the current study, we compared the gathering and use of local medicinal plants in the Upper Sangone Valley, Western Italian Alps, Piedmont (NW Italy) as described in a field study conducted in the mid-seventies and published in 1977 and those arising from field research that we conducted in the spring of 2015 and 2018, during which time ethnobotanical and ethnomycological information concerning both folk medicinal and wild food uses was obtained via 47 in-depth open and semi-structured interviews with community members.

**Results:**

In total, one hundred thirty folk taxa represent the past and present medicinal and wild food plant/mushroom heritage of the Sangone Valley: 26 herbal taxa were recorded 40 years ago only; 68 herbal and wild food taxa have been recorded in the current study only; and 36 herbal taxa have been continuously used during the last 40 years. There were no remarkable quantitative differences between the two diachronic medico-ethnobotanical datasets, but the qualitative differences were substantial. The gathering and use of some medicinal plants growing in meadows, forests and higher mountain environments (i.e. *Arctostaphylos*, *Filipendula*, *Hepatica*, *Larix*, *Laserptium*, *Picea*, *Polygonatum*, *Primula*, *Tussilago* and *Veronica* spp.) disappeared, whereas the collection of plant genera growing in more anthropogenic environments or possibly promoted via popular books and media has been newly introduced (i.e. *Aloysia*, *Apium*, *Brassica*, *Crataegus*, *Epilobium*, *Fumaria*, *Geranium*, *Juniperus*, *Melissa*, *Rubus*, *Rumex*, *Sedum*, *Silybum*, *Taraxacum* and *Vaccinium* spp.).

**Conclusion:**

The findings show a renegotiation of the *situativity* that for centuries forged the embeddedness of local communities in their natural environments, probably heavily informed in the past by prevalent pastoralist and forest-centred activities and thus by a deeper knowledge of higher mountain and forest environments. The re-arrangement of a more domestic and more “globalized” herbal knowledge system was possibly inspired by new urban residents, who started to populate the valley at the end of the Seventies, when the original inhabitants abandoned their homes for the urban centres of the Piedmontese plain. The current study suggests that future directions of ethnobiological research should more carefully look at the *adaptive capacity* of LEK systems.

## Introduction

In the past decades, local environmental knowledge (LEK) of mountain communities has increasingly been at the centre of important field researches and public debates as it is nowadays widely considered a precious resource for sustainable development [1–6]. The value of LEK, however, has often been perceived in opposition to modernity which draws from the peripherality of the communities and their distance from cities, the main perceived catalyst centres of “modernity”. This assumption, though, has reiterated a long-standing dichotomic model that represents part of the problematic scientific debate which views the LEK of marginal communities in opposition to modernity [7, 8]. The relationship between LEK and modernity has often been described with binary conceptualizations, such as the science of the concrete vs. “the sciences” [9], tacit knowledge vs. scientific knowledge [10], oral knowledge vs. written knowledge [11], Indigenous Knowledge vs. Western knowledge [12] and Traditional Knowledge vs. modern knowledge [13].

“Situated” local ecological knowledge (LEK) refers to a system of knowledge, practices and beliefs that is profoundly embedded within a given socio-ecological *endroit* [14]. Lauer and Aswani [15] described how LEK in the Western Salomon Islands overcame the distinction between cognitive aspects and other modalities of knowing and how it is deeply intertwined with everyday ecological practices. Ecological knowing and practicing is more than a mere, even complex, system and Whyte [16] argued that this LEK is actually to be seen as a collaborative concept and that scientists, environmentalists and local communities should try to create long-term processes that allow the different implications of approaches to knowledge in relation to stewardship goals to be responsibly considered. In this perspective, the concept of *situated* LEK has become a useful tool in regional planning linked, in particular, with rural development [17]. More broadly, the debate about *situated* knowledge, however, highlights the dynamicity of local knowledge and examines the possible drivers of change in order to support local communities and environmental preservation.

In a recent review that assessed the global literature and analysed the drivers of various types of LEK transformations [18], the authors postulated that the impoverishment of LEK may be driven by globalization, modernization, and market integration. Moreover, they highlighted the unsymmetrical loss of LEK, with losses being more markedly recorded in medicinal and botanical domains.

Changes in LEK measured as knowledge and practices concerning the ecosystem have been the subject of a few studies in recent years [19, 20]. Liu et al. [19] described the industry-, government- and culture-driven changes of LEK of Tao fly fishing culture in Taiwan and found that various external factors have contributed to LEK changes, such as inappropriate government policies, modern science and technology, education and the market economy. Duenn et al. [20] studied the perception of invasive alien species among Rabari pastoralists from Gujarat, India, and the results showed that alien species invasion was not perceived as a major problem; the authors postulated that the adaptive capacity of LEK systems and a slow rate of environmental change directly attributable to alien species may explain this apparent “paradox”.

However, changes in folk botanical knowledge, assessed using comparable research methods (ethnography-based techniques, e.g. face-to-face interviews), remain poorly investigated. Some diachronic comparisons in ethnobotany have been attempted, with interesting results, in Eastern Europe [21–26] and, more recently, in Southern Europe [27], while beyond Europe this has been more sporadically done [28, 29]. In particular, in Poland, a comprehensive historical study showed that LEK linked to the gathering of wild green vegetables has gradually decreased, mainly due to replacement by a few cultivated vegetables; this process started in the nineteenth century, but became more pronounced during the twentieth century [22]. Moreover, a review focusing on wild food plant gathering in Europe [30] has underlined not only the decline of “traditional” foraging but also the remarkable emergence of a new, “recreational” foraging, practiced by urban classes, often popularized by trained botanists, organic farmers, chefs, and food activists, and whose real impact among the majority of the population in Western societies has still to be properly assessed. Schunko et al. [31] well demonstrated a decade ago that the motivation of the resurgent popularity of wild plant gathering among organic farmers in Austria is to be attributed to an “internalization” of motivations, i.e. the pleasure of the activity itself, while diverse authors in Europe ([32], and chapters therein [33]) discussed how the resilience or resurgence of foraging may be linked to the increasing attention communities and citizens pay to their local/regional cultural identities and to the perceived healthy properties of the wild food plants.

On the herbal ethnobotanical side, changes in medicinal plant use in Estonia between 1888 and 1994 showed that the use of garlic, hose chestnuts and mint increased, whereas the use of barley, orchids, *Paris quadrifolia* and *Briza media* significantly decreased [23], thus supporting the idea that during the past century the utilization of plants depending on human influence was remarkably amplified.

On the other hand, the lack of extensive literature providing diachronic ethnobotanical data is due to the fact that the few field studies carried out decades ago were often conducted by researchers (primarily folklorists or local historians) who were not able to present reliable botanical identifications or, more rarely, by biologists who had never been exposed to the social sciences. We believe that these limitations can greatly affect the validity of comparative works much more than is generally assumed by many researchers. However, in one study that was conducted in the mid-seventies (most likely in 1976) in the Upper Sangone Valley, a tiny valley of the Western Italian Alps [34], researchers documented folk herbal practices via face-to-face interviews with locals. We therefore decided to conduct the current field research in exactly the same area, in order to analyse the ethnobotanical picture 40 years later.

The present paper provides a contribution to the aforementioned debate by exploring the changes in LEK in a Western Alpine valley during the past 40 years, using as a proxy a comparison between the medicinal plants gathered and used by the local community in the mid-seventies and those gathered and used in the years 2015–2018.

The specific objectives of this study therefore were:

- to investigate the *current* ethnobotanical heritage (including both wild food and herbal plant and mushroom uses) of the Upper Sangone Valley;
- to compare the recorded herbal uses with those recorded forty years earlier;
- to interpret possible differences in both ecological and/or cultural terms and to discuss the diachronic transformation of local plant knowledge.

## Materials and methods

### Selection of the ethnobotanical literature

A decade ago, our research group started an investigation on the historical ethnobotanical literature of Italy from 1884 to the 1980s [35, 36] and selected those works which included both reliable botanical identifications (i.e. works indicating voucher specimens, or studies conducted by academic botanists or, even better, botanical taxonomists) and a clear description that the ethnobotanical information was collected via face-to-face interviews. At the end of this months-long work, we obtained no more than a dozen field studies conducted between 1955 and 1980 in NW, NE and Central Italy, which could be considered appropriate for diachronic comparison. Among this restricted group, one study conducted in the mid-seventies in the Western Alps emerged [34]. This particular field study was conducted in the Upper Sangone Valley and subsequently published in an Italian hospital pharmacy journal in 1977. The work was conducted, without the collection of vouchers, by academic botanists via face-to-face interviews with locals, focusing specifically on local *wild and cultivated medicinal plants* gathered and used in domestic herbal medicine or as a medicinal food (i.e. herbal liqueurs and other food preparations consumed with the express purpose of treating certain illnesses). The work did not document, however, any frequency of quotation, or frequency of use, of the cited medicinal plants, or details regarding the areas in which the plants were collected.

### Current field study

**Fig. 1:**
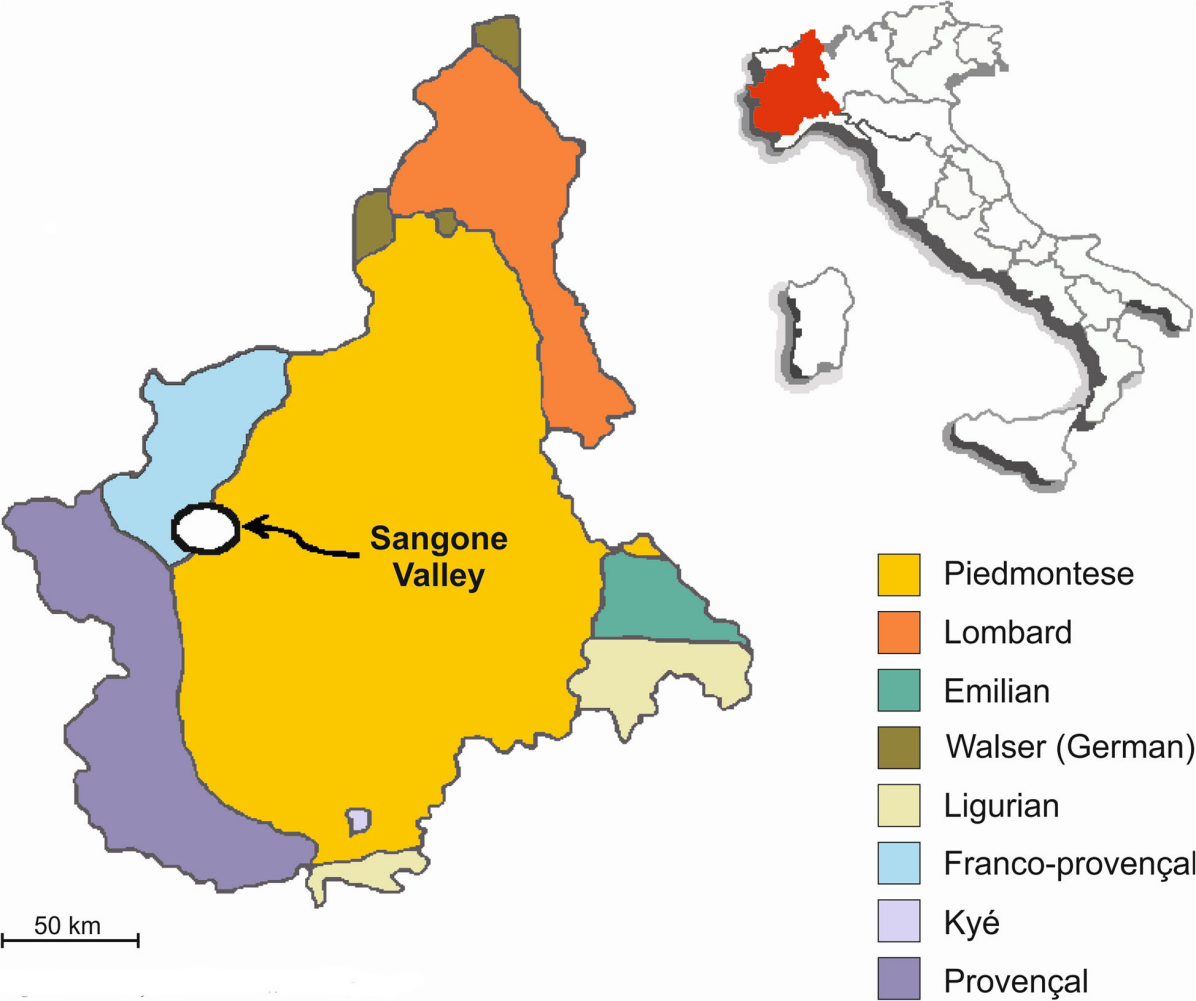
The study area within the lingustic map of Piedmont, NW Italy

The current field study was conducted in the same area where the aforementioned study was conducted in the Seventies: the Upper Sangone Valley, Cottian Alps, Piedmont, NW Italy (Fig. 1). The following small villages were visited: Alpe Palè (N 45° 03′ 56″ E 7° 13′ 32″, Coazze Muncipality, 1356 m a.s.l.; approximately 10 inhabitants); Borgata Tora (N 45° 00′ 33″ E 7° 18′ 11″, Giaveno Municipality, 994 m a.s.l.; approximately 20 inhabitants); Cervelli (N 45° 03′ 13″ E 7° 16′ 06″, Coazze Municipality, 881 m a.s.l., approximately 40 inhabitants); Colle Braida (N 45° 04′ 59″ E 7° 19′ 59″, Valgioie Municipality, 1008 m a.s.l.; 10 inhabitants); Mollar dei Franchi (N 45° 01′ 28″ E 7° 20′ 17″, Giaveno Municipality, 563 m a.s.l.; 50 inhabitants); Roccette (N 45° 01′ 37″ E 7° 17′ 45″, Giaveno Municipality, 780 m a.s.l.; 60 inhabitants); and Prè Fieul (N 45° 01′ 42″ E 7° 16′ 15″, Giaveno Municipality, 1263 m a.s.l.; approximately 20 inhabitants). The main centre of Coazze (N 45° 03′ 07″ E 7° 18′ 02″, 750 m a.s.l., approximately 2700 inhabitants) was also included, since a few inhabitants maintain links to pastures in higher valleys.

The field study was carried out by the two authors with the help of some trained students (see acknowledgments) in the spring of 2015 and 2018. In-depth open and semi-structured interviews were conducted with community members (*n* = 47, average age 65; 21 interviews were conducted in 2015 and 26 in 2018) in the aforementioned eight settlements. Participants were selected using snowball sampling techniques, focusing specifically on those elderly community-members (e.g. farmers, housewives) who still have connections to an agro-pastoral way of life. Informants were asked about current and recent past gathering and use of (a) local wild food plants; (b) wild and domesticated medicinal and veterinary plants; and (c) mushrooms for food and/or medicine. The second category (b) included both proper medicinal plants and also plant-based medicinal foods (i.e. herbal liqueurs and other food preparations consumed with the express purpose of treating certain illnesses).

Specifically, study participants were asked about the local plant name(s) and plant part(s) used, as well as specific details about their manipulation/preparation and actual herbal or food use(s). Interviews were conducted in Italian. Prior informed consent was always verbally obtained prior to the interviews and researchers adhered to the ethical guidelines of the International Society of Ethnobiology [37]. During the interviews, informants were always asked to show the quoted plants or, at least, to describe them. Botanical excursions both around the house and in the mountains were additionally conducted in order to collect botanical specimens, which are deposited at the Herbarium of the University of Gastronomic Sciences. Taxonomic identifications were carried out by the first author according to the Flora of Italy [38], while botanical nomenclature was later standardized using The Plant List database [39]. Family assignments follow the Angiosperm Phylogeny Group IV system [40] and mushroom nomenclature follows the Index Fungorum database [41].

### Study area

The surrounding landscape is typical of pre-Alpine and Alpine habitats. Chestnut and beech tree forests dominate up to 1000 m, while higher elevations are covered by fir, pine and birch forests, as well as grasslands with rhododendron and blueberry bushes at over 2500 m a.s.l. The upper part of the territory is occupied by Orsiera-Rocciavrè Natural Park, a protected area of approximately 10,000 ha, established by the region of Piedmont in 1980. The park extends into the Northern Cottian Alps, the boundaries of which run, on average, at an altitude of 1400 m a.s.l., and the most important mountain peaks included within the park reach nearly 3000 m a.s.l. The altitude of thermal zero in winter is 1700 m a.s.l. The territory is characterized by relatively low rainfall, which is among the lowest in NW Italy. On average, maximum rainfall is in May and October and the minimum is in January and July. The geomorphological characteristics of the park territory allow many animal species to find suitable habitat, among them hares, rock ptarmigans, marmots, chamois and mouflons.

As in other valleys in the Western Alps [42–45], the traditional economy of the communities in the Sangone Valley relied on a multifunctional agricultural model based on small family farms, which combined the cultivation of cereals (mainly barley, rye, and maize) with that of vegetables (such as cabbages, carrots, onions, potatoes, onions, turnips) and fruits (apples, pears). In addition, and more importantly, it also included cattle, sheep and goat pastoralism, which supported local dairy production, while hunting and gathering practices (which included the collection of chestnuts, mushrooms and herbs used for both food and medicine) were mainly carried out in forests and higher pastures. This model was still followed in the early decades of the twentieth century [46]. Despite the importance of agriculture, the manufacturing industry has played a key role in the valley since the late nineteenth century. The Sangone Valley is located on one of the main transit routes between Italy and France [47], and its municipalities are easily accessible from Turin, one of the most important industrial cities in Italy. This allowed rapid development of the manufacturing industry in the valley beginning in the nineteenth century [48–50] and fostered the emergence of a mixed household economy that linked wage-earning to multifunctional farming activities. Moreover, at the beginning of the twentieth century, the valley became one of the first destinations for Alpine tourism [51], primary associated with trekking and cross-country skiing, which further complexified the local economy. As in the rest of the Alpine region [52], the post-WWII period marked the decline of agriculture as an economic sector of the valley, with the reduction of cultivated land in the valley [53], as well as specialization of agricultural production (e.g. apples, pears). While in the 1960s and 1970s, the valley experienced fast industrial development, in the following decades, like the rest of the region [54], the economy of the Sangone Valley opened to the service sector, accelerating the agricultural decline. In this transformation, tourism has played a vital role, expanding its reach and encompassing initiatives linked to the promotion of local cultural and gastronomic heritage [55, 56].

The local language of the Upper Sangone Valley, referred to by locals as *patois*, is Franco-Provençal (also known as Francoprovençal, Arpitan or Romand), which is mainly used only in the domestic arena by elderly community members; both standard Italian and the Piedmontese dialect are largely dominant among the middle and younger generations and in all communication with external visitors. Franco-Provençal represents a dialect group within the Gallo-Romance language family, which is also spoken in East and Central France, Western Switzerland, NW Italy, and in two tiny enclaves in SE Italy. Its current use within the domestic arena is decreasing, although some organizations are attempting to preserve this linguistic heritage through cultural events, educational platforms and some publishing activities [57].

### Data analysis

The medicinal botanical data recorded in 2015 and 2018 were tabulated and compared with those arising from the medical-ethnobotanical fieldwork that was conducted in the mid-seventies in the same area [34] via a matrix describing the plant genera traditionally gathered and used for medicinal purposes nowadays and/or 40 years ago, which was then visualised in a Venn diagram.

Additionally, the recorded plant reports were qualitatively compared with the most comprehensive worldwide wild food plant compendia [58, 59], the Italian medical ethnobotanical database [60], an ethnobotanical survey carried out in a nearby valley one decade ago [61], and a few recent, sporadic ethnobotanical studies conducted on the Italian side of the Alps [62–65], in order to point out possible novel reports (i.e. reports not previously recorded in the Italian ethnobotanical literature).

Moreover, the most important written popular sources of herbal knowledge published in Italy from the 1970s until now were also considered [5, 66–72], in order to qualitatively assess the possible correspondence between “new” recorded herbal customs and their recurrent presence in this literature.

## Results

### Plants and mushrooms for food and medicine in the Sangone Valley

In total, one hundred thirty *folk taxa* (i.e. taxa linked to a single folk plant/mushroom name) were recorded, representing the past and present medicinal and food heritage of the Sangone Valley (Table 1):

- 26 taxa used as medicine were recorded 40 years ago only;
- 68 taxa were recorded in the current study only—as locally gathered plants/mushrooms for food only (33 taxa), for medicinal uses only (23 taxa) or for both (12 taxa);
- 36 taxa have been continuously used during the last 40 years.

**Table 1:** Local wild food and medicinal plants gathered and used in the study area

Gathered botanical taxon/taxa, family/-ies and voucher specimen code(s)	Local plant name(s)	Used parts	Folk use recorded in 1976 (preparation)	Folk use recorded in the current field study (preparation)	Frequency of use in the current field study
*Acer pseudoplatanus* L., Aceraceae, UNISGSANG18001	Piè	Leaves		FOO: wrapping for butter; VET: fodder for goats	N/A (remembered from the past)
*Achillea herba*-*rotta* All., *A*. *millefolium* L., and possibly *A*. *nana* L., Asteraceae, UNISGSANG15001 (*A. herba-rotta*) and UNISGSANG18005 (*A*. *millefolium*)	Marmin	Whole plant	HER: diuretic, febrifuge (tea)	FOO/HER: liqueurs (considered diuretic); HER: diuretic (tea); anti-hematomas and anti-bruises (poultice with lard and vinegar)	Very common
*Adianthum hispidulum *Sw., Pteridaceae, UNISGSANG18045	Fugèt	Aerial parts		HER: enhancing blood circulation, emmenagogue (tea)	Rare
*Alcea rosea* L., Malvaceae			HER: anti-kidney stones (decoction)		
*Alchemilla xanthochlora *Rothm., Rosaceae	Cuculin	Leaves		HER: anti-dysmenorrhea (external washes)	Rare
*Allium cepa* L., Amaryllidaceae	Siola	Bulbs		HER: bechic (tea), digestive (broth), anti-inflammatory and panacea (cooked)	Very common
*Allium sativum* L., Amaryllidaceae	Ai	Bulbs	HER: vermifuge (also in necklaces to be worn during the night)	HER: hypotensive (food); vermifuge in children (slice of bread rubbed with garlic)	Fairly common
*Allium schoenoprasum* L., Amaryllidaceae	Erba cipollina	Leaves		FOO: seasoning	Rare
*Allium ursinum* L., Amaryllidaceae	Aiet	Leaves		FOO: salads, soups	Rare
*Alnus incana* (L.) Moench, Betulaceae, UNISGSANG15002	Verna	Leaves		HER: anti-back pain (compress of dried leaves)	Rare
*Aloysia citrodora* Palau, Verbenaceae, UNISGSANG18057	Limonaria	Leaves		HER: digestive (tea)	Rare
*Althaea officinalis* L., Malvaceae, UNISGSANG15003	Redaui	Roots	HER: anti-kidney stones (decoction)	HER: anti-toothache in children (tea made from fresh roots)	Rare
*Angelica archangelica L*. and *A*. *sylvestris* L., Apiaceae	Angelica	Roots and aerial parts	HER: digestive (liqueur made from the roots)	HER: anti-carbuncles (compress of aerial parts)	Rare
*Apium graveolens* L., Apiaceae	Serli	Leaves		HER: male aphrodisiac (consumed)	Rare
*Arctium lappa* L., Asteraceae, UNISGSANG15004	Cicapuoi	Leaves	HER: bechic (decoction)	HER: hepato-protector (decoction)	Rare
*Arctostaphylos uva*-*ursi* (L.) Spreng., Ericaceae		Leaves	HER: anti-urinary tract infections (decoction)		
*Armillaria mellea* (Vahl.) P.Kumm., Physalacriaceae	Famiole	Fruiting body		FOO: pickled in oil	Very common
*Arnica montana* L., Asteraceae, UNISGSANG18006, UNISGSANG15005	Arnica, Bertolica	Flowers		HER: for treating many skin inflammations, anti-bruises, anti-rheumatic (oleolite or alcoholic macerate, externally applied); anti-flu (inhaled)	Very common
*Artemisa absinthium* L., Asteraceae, UNISGSANG15007	Erba banci, Incens	Leaves and flowers	HER: vermifuge (decoction)	VET: enhancing placenta expulsion (fodder in cows)	Rare
*Artemisia genipi* Weber ex Stechm. and possibly *A*. *glacialis* L. and *A*. *umbelliformis* Lam., Asteraceae, UNISGSANG15006 (*A*. *genipi*)	Genepì (*A*. *genipi*), Genepì fumela (A. *glacialis* and *umbelliformis*)	Aerial parts	HER: digestive (tea)	FOO/HER: liqueurs (considered digestive); HER: digestive (tea)	Fairly common
*Aruncus dioicus* (Walter) Fernald., Rosaceae	Spars servai	Shoots		FOO: boiled	Fairly common
*Betula pendula* Roth, Betulaceae, UNISGSANG18010	Bès	Sap		HER: blood depurative (drunk)	Rare
*Boletus aureus* Schaeff., Boletaceae	Moru	Fruiting body		FOO: salads, sautéed, fried, pickled	Very common
*Boletus edulis* Bull., Boletaceae	Bouloi	Fruiting body		FOO: salads, sautéed, fried, pickled	Very common
*Brassica oleracea* L. var. *sabauda*, Brassicaceae	Coi	Leaves		HER: anti-bruises (topically applied)	Rare
*Brassica rapa* L., Brassicaceae	Rava	Roots	HER: bechic (also for whooping cough; slices of the root mixed with honey and left to macerate 3 days)	FOO: external “peel” dried and cooked in milk when vegetables were scarce in the spring	N/A (remembered from the past)
*Calluna vulgaris* (L.) Hull, Ericaceae, UNISGSANG15009	Bruri	Leaves		HER: anti-diarrhoeic (decoction); VET: fodder for rabbits	Rare
*Carlina acanthifolia* All., Asteraceae, UNISGSANG18007	Cardun, Pugnu	Flowers and flower receptacles		FOO: snack (flower receptacles); FOO/HER: digestive (liqueur made from the flowers)	Fairly common (FOO); rare (FOO/HER)
*Carum carvi* L., Apiaceae, UNISGSANG15010	Cumino, Cümmel	Fruits		FOO/HER: liqueur (considered digestive)	Rare
*Castanea sativa* Mill., Fagaceae, UNISGSANG18022	Castagn	Fruits		FOO: soups with milk and potatoes, roasted, jams, sweets, liqueurs	Very common
*Ceterach officinarum* Willd., Aspleniaceae		Aerial parts	HER: diuretic, vermifuge (decoction, taste sometimes improved with a few mint leaves)		
*Cetraria islandica* (L.) Ach., Parmeliaceae, UNISGSANG15011	Lica	Thallus (traditionally gathered in fields over 2,000 m a.s.l. on the 2nd Sunday of July)	HER: bechic and demulcent (decoction, sometimes together with lime tree flowers)	HER: bechic and anti-inflammatory of the respiratory tract (decoction or syrup)	Fairly common
*Chantarellus cibarius* Fr., Cantharellaceae	Garitula	Fruiting body		FOO: soups, sautéed, fried	Very common
*Chelidonius majus* L., Papaveraceae	Erba di poret	Latex	HER: anti-warts (topically applied)		
*Chenopodium bonus*-*henricus* L., Amaranthaceae, UNISGSANG18014	Spinas sarvai	Leaves		FOO: cooked	Fairly common
*Cichorium intybus* L., Asteraceae	Cicoria	Leaves	HER: depurative (decoction)		
*Crataegus monogyna* Jacq., Rosaceae, UNISGSANG15012	Bosu	Leaves and flowers		HER: tranquilizing, hypotensive (tea)	Rare
*Cynodon dactylon* (L.) Pers., Poaceae, UNISGSANG18037, UNISGSANG15013	Gramon, Grmon	Whole plant	HER: diuretic (tea, often mixed with corn stigmas and bearberry leaves)	HER: diuretic (tea)	Fairly common
*Daphne mezereum* L., Thymelaeceae		Roots	VET: purgative (tea)		
*Eleagnus rhamnoides* (L.) A.Nelson		Fruits	HER: astringent and anti-hemorrhagic (crushed fruits, consumed or topically applied)		
*Epilobium angustifolium* L., Onagraceae, UNISGSANG15014	Epilobio	Leaves		HER: anti-cancer	Rare
*Equisetum arvense* L., Equisetaceae, UNISGSANG18015	Coa d’aval Erba cavaligna Erba cavallina	Aerial parts	HER: diuretic, improving blood circulation (decoction)	HER: diuretic, improving joints	Rare
*Erica arborea* L., Ericaceae, UNISGSANG15016	Erica	Flowering tops		HER: diuretic (tea)	Rare
*Filipendula ulmaria* (L.) Maxim., Rosaceae		Flowering tops	HER: diuretic (tea)		
*Fistulina hepatica* (Schaeff.) With., Fistulinaceae	Lenghe	Fruiting body		FOO: salads	Fairly common
*Fragaria vesca* L., Rosaceae, UNISGSANG18047	Frola	Fruits		FOO: snack; FOO/HER: for treating intestinal discomforts (consumed)	Very common (FOO); rare (FOO/HER)
*Fumaria officinalis* L., Papaveraceae	Fumaria	Leaves		HER: for treating eye inflammations, decoction (topically applied)	Rare
*Gentiana acaulis* L. and *G*. *verna* L., Gentianaceae, UNISGSANG18025 and UNISGSANG15018, respectively	Cücüc, Giansanela	Roots and whole plant (*G*. *acaulis*);Flower buds (*G*. *verna*)	FOO/HER: aromatized wines made from the roots of *G*. *acaulis* (considered digestive)	FOO/HER: aromatized wine and liqueurs made from the whole plant of *G*. *acaulis* or the flower buds of *G*. *verna* (considered appetizing, digestive, and reconstituent)	Fairly common
*Gentiana lutea* L., Gentianaceae, UNISGSANG18027	Giansana Giansena	Roots	FOO/HER: aromatized wines (considered digestive and hepato- protector)	FOO/HER: aromatized (white) wines, sometimes adding lemon peels (considered appetizing, digestive, and reconstituent); VET: appetizing and digestive (cows)	Very common
*Geranium robertianum* L., Geraniaceae, UNISGSANG15019	Erba roberta	Flowers		HER: to treat menstrual pains (decoction)	Rare
*Grifola frondosa* (Dicks.) Gray, Grifolaceae	Mutun	Fruiting body		FOO: pickled in oil	Fairly common
*Hedera helix* L., Araliaceae	Edera	Leaves	HER: wounds (often with St. John’s Wort oleolite)	HER: for treating cradle cap (tea, externally applied)	Rare
*Helminthoteca echioides* (L.) Holub, Asteraceae	Patacun, Scagnet	Leaves		FOO: soups; VET: fodder for rabbits	Fairly common
*Hepatica nobilis* Mill., Ranunculaceae		Leaves	HER: anti-horsefly bites (crushed leaves topically applied)		
*Humulus lupulus* L., Cannabaceae, UNISGSANG15020, UNISGSANG18012	Luvertin,Lüvertin	Shoots		FOO: risotto, omelettes	Very common
*Hypericum perforatum* L., Hypericaceae, UNISGSANG15021, UNISGSANG18027	Erba d’San Giuan, Milapertus, Stafurà, Trafurera Tucram	Aerial parts and flowers	FOO/HER: liqueurs made from the leaves (considered digestive and intestinal spasmolytic);digestive, hepato-protector, cholagogue (decoction of the flowers); for treating all skin inflammations (including burns and wounds; oleolite, often extracted in walnut oil and mixed with ivy leaves, topically applied); VET: for treating leg infections in calves, sheep, and goats (oleolite mixed with bran topically applied)	FOO/HER: aromatized wines made from the whole aerial parts (considered tranquilizing), liqueurs made from the flowers (considered tranquilizing and anti-cholesterolemic); HER: anti-burns and anti-wounds (oleolite, topically applied); anti-stomach ache and anti-cystitis (oleolite, drunk)	Very common
*Hyssopus officinalis* L., Lamiaceae	Issop	Flowers	HER: bechic (tea)		
*Juglans regia* L., Juglandaceae, UNISGSANG1828	Nus	Unripe fruits and kernels		FOO/HER: liqueurs made from unripe fruits (considered digestive); sweets, salads with garlic, vinegar, and salt (kernels)	Very common
*Juniperus communis* L., Cupressaceae, UNISGSANG15022	Genevru	Branches, bark and galbules		FOO: roasted meat seasoning (galbules); grappa seasoning (branches); VET: general sickness (galbules as fodder for cows and bark as fodder for rabbits)	Very common (FOO); rare (VET)
*Laburnum anagyroides *L., Fabaceae, UNISGSANG18020	Aburn	Branches, leaves and flowers		VET: insect repellent (hang in the hen-house)	Rare
*Lapsana communis* L., Asteraceae, UNISGSANG18060	Burasa russa, Scina rusa	Leaves		FOO: soups	Rare
*Larix decidua* Mill., Pinaceae	Pin	Resin and young shoots	HER: muscular pains and arthritis (oleolite, externally applied); bechic (decoction)		
*Laserpitium siler* L., Apiaceae	Apia	Leaves	HER: anti-bruises (crushed leaves mixed with pork fat and topically applied)		
*Leccinum scabrum* (Bull.) Gray, Boletaceae	Crava			FOO: sautéed, fried	Fairly common
*Malva sylvestris* L., Malvaceae, UNISGSANG15025, UNISGSANG18033	Malva, Marva, Riondela	Leaves and flowers	HER: tranquilizing, anti-neuralgic, bechic (tea)	FOO: soups (young leaves); HER: digestive, anti-inflammatory, anti-cystitis (tea); for treating eye inflammation (anti-stye; tea, externally applied)	Very common
*Matricaria chamomilla* L., Asteraceae	Canamia	Flowering tops	HER: tranquilizing, digestive (tea)	HER: tranquilizing (tea); for treating eye and ear inflammations (oleolite, externally applied); to treat abdominal pains (hot compress applied to the belly)	Very common
*Melissa officinalis* L., Lamiaceae, UNISGSANG15026, UNISGSANG18029	Limonaria, Mlissa	Leaves		FOO: seasoning; HER: digestive, emmenagogue (tea)	Very common
*Mentha spicata* L. and possibly other *Mentha* spp., Lamiaceae, UNISGSANG18030	Menta	Leaves	HER: digestive (tea)	FOO/HER: liqueurs (considered digestive); HER: digestive (tea); VET: anti-lice in hens	Very common (vet use rare)
*Myosotis sylvatica* Hoffm. (Boraginaceae), UNISGSANG18011	Non ti scordar di me	Flowers		FOO: salads; HER: for treating eye inflammations (topically applied)	Rare
*Nasturtium officinale* R. Br., Brassicaceae, UNISGSANG18012	Grisun	Aerial parts		FOO: salads (considered blood depurative)	Fairly common
*Neoboletus luridiformis* (Rostk.) Gelardi, Simonini & Vizzini, Boletaceae	Fre	Fruiting body		FOO: cooked	Rare
*Oxalis acetosella* L., Oxalidaceae, UNISSANG15027	Trampis, Trampin			FOO: omelettes	Fairly common
*Papaver rhoeas* L., Papaveraceae	Papaver	Petals	HER: tranquilizing, febrifuge (tea)		
*Parietaria officinalis* L., Urticaceae		Aerialparts	HER: anti-kidney stones (decoction)	HER: diuretic, anti-kidney stones, for treating shingles (decoction)	Fairly common
*Persicaria bistorta* L. Samp., Polygonaceae, UNISGSANG18041	Lenghe buine	Leaves		FOO: cooked; VET: fodder for goats	Fairly common
*Picea abies* (L.) H.Karst., Pinaceae	Sapin	Resin and young shoots	HER: muscular pains and arthritis (oleolite, externally applied); bechic (decoction)		
*Pinguicula* *grandiflora* Lam., and possibly other *Pinguicola* spp., Lentibulariaceae, UNISSANG15028 (*P*. *grandiflora*)	Viola tajarda	Leaves	HER: anti-bruises (chopped leaves mixed with lard and topically applied)	HER: vulnerary (topically applied)	Rare
*Pinus mugo* Turra and possibly other *Pinus* spp.,. Pinaceae, UNISGSANG15029 (*P*. *mugo*)	Pin	Resin and young shoots	HER: muscular pains and arthritis (oleolite, externally applied); bechic (decoction)	FOO/HER: syrups, liqueurs (considered bechic)	Rare
*Plantago coronopus* L., Plantaginaceae, UNISGSANG15030	Piota de galign	Leaves		FOO: soups	Fairly common
*Plantago lanceolata* L., Plantaginaceae, UNISGSANG15031, UNISGSANG18036	Cüjet, Erba dii canarin, Urie du giari	Leaves	HER: anti-carbuncles, (crushed leaves, topically applied)	FOO: soups; HER: anti-carbuncles, anti-haemorrhoids, (compress, externally applied)	Fairly common
*Plantago major* L., Plantaginaceae, UNISGSANG15037	Piantagn	Leaves		FOO: soups; HER: diuretic (leaves)	Rare
*Polygonatum multiflorum* (L.) All., Asparagaceae		Rhizomes	HER: anti-carbuncles (externally applied)		
*Polypodium vulgare* L., Polypodiaceae, UNISGSANG15032, UNISGSANG18043	Argalisia	Rhizomes		FOO: snack	Rare
*Potentilla anserina* L., Rosaceae, UNISGSANG15033	Erb del set virtü	Rhizomes		HER: anti-diarrheic, febrifuge, anti-stomach ache, bechic (decoction)	Fairly common
*Primula vulgaris* Huds. and possibly *P. veris* L., Primulaceae, UNISGSANG15034, UNISSANG18044 (*P*. *vulgaris*)	Piumbera, Ptrine	Flowers and roots	HER: bechic (decoction of the roots)	FOO: soups (flowers)	Fairly common
*Prunus avium* L., Rosaceae	Cerese, Cerse büshas	Fruit peduncles and fruits	HER: febrifuge (tea of the peduncles)	FOO: snack, jams, preserved (fruits); HER: anti-inflammatory, diuretic (decoction of the peduncles)	Very common
*Prunus cerasus* L., Rosaceae	Griutera	Fruits		FOO: snack	Very common
*Prunus domestica* L., Rosaceae	Bergna	Kernels	HER: vermifuge (crushed kernels mixed with honey and consumed)		
*Prunus padus* L., Rosaceae		Bark	HER: febrifuge (tea)		
*Pulmonaria officinalis* L., Boraginaceae		Leaves	HER: bechic (tea)		
*Quercus petraea* (Matt.) Liebl. and possibly *Q*. *robur* L., Fagaceae, UNISGSANG18024 (*Q*. *petraea*)	Rol, Rul	Leaves, bark and acorns	VET: astringent (decoction of bark and acorns for calves)	VET: galactagogue (dried leaves as fodder for goats)	Rare
*Ranunculus acris* L. Ranunculaceae, UNISGSANG15035	Ranuncolo	Flowers		HER: rubefacient (applied under the feet to artificially generate a rash for avoiding compulsory military service)	N/A (remembered from the past)
*Rhododendron hirsitum* L. and possibly *R. ferrugineum*, Ericaceae, UNISGSANG15036 (*R*. *hirsitum*)	Brunsai	Flowers	HER: bechic (decoction); anti-kidney stones (tea)	HER: diuretic (decoction)	Rare
*Robinia pseudoacacia* L.Fabaceae, UNISGSANG18021	Gasia	Inflorescences		FOO: deep fried	Fairly common
*Rosa canina* L., Rosaceae, UNISGSANG18050	Gratacul, Gratacü	Pseudo-fruits	HER: astringent (tea)	FOO: jams, liqueurs;HER: diuretic (decoction)	Fairly common
*Rubus idaeus* L., Rosaceae, UNISGSANG18051	Àmpula	Fruits		FOO: snack, jams, liqueurs	Very common
*Rubus ulmifolius* Schott., Rosaceae, UNISSANG15037, UNISGSANG18052	Runza	Young shoots, unripe and ripe fruits		FOO: jams (fruits);soups (young shoots); HER: anti-hoarseness (gargles of a decoction of the unripe fruits); anti-diarrheic (decoction of the young shoots); vulnerary and anti-septic (alcoholic macerate of the young shoots, externally applied)	Fairly common
*Rumex acetosa* L., Polygonaceae, UNISGSANG15038, UNISGSANG18042	Esileu, Isiule	Leaves		FOO: salads, omelettes, sauce (mixed with milk and ghees) for accompanying polenta; HER: diuretic (tea)	Fairly common
*Rumex alpinus* L., Polygonaceae, UNISGSANG15039	Ariei, Lapas, Rebarbar d’montagna	Leaves, inflorescences and roots	HER: digestive (tea of the leaves and inflorescences)	FOO: soups,wrapping for butter (leaves); HER: digestive (root decoction)	Fairly common (FOO; wrapping for butter remembered from the past); rare (HER)
*Ruta graveolens* L., Rutaceae	Ruta	Leaves		FOO: grappa seasoning	Fairly common
*Salix caprea* L., Salicaceae	Sales	Leaves and bark	HER: anti-callus (crushed, externally applied)		
*Salvia officinalis* L., Lamiaceae, UNISGSANG18031	Sarvia	Leaves	HER: digestive (decoction and liqueur)	HER: tranquilizing, digestive, reconstituent (decoction, not to be used during pregnancy); anti-septic (washes)	Very common(old local saying: “Se la fumna a savèisa la virtü d’la salvia, a saria mai malavia” = “If a woman knew the virtues of sage, she would never be ill”)
*Sambucus nigra* L. and *S*. *racemosa* L., Adoxaceae, UNISSANG15040 (*S. nigra*)	Sambur	Young branches, flowers and fruits	FOO/HER: aromatized wines made from the flowers and fruits (considered depurative);HER: anti-rheumatic (oleolite of the flowers, externally applied)	FOO: jams (fruits or flowers), soups (flowers); HER: anti-herpes, anti-burns, for treating skin inflammations, anti-haemorrhoids (poultice mixing bee wax and young branches); anti-toothache (flowers, externally applied); diuretic and febrifuge (decoction of the flowers); anti-toothache and anti-rheumatic (decoction of the flowers in milk, externally applied)	Very common
*Scabiosa columbaria* L., Caryophyllaceae, UNISGSANG15056, UNISGSANAG18059	Gialina grasa, Gialina grasa	Whorls		FOO: soups	Rare
*Sedum telephium* L., Crassulaceae, UNISSANG15043	Erba dei calli	Aerial parts		HER: anti-callus (oleolite, externally applied)	Rare
*Sempervivum* sp., Crassulaceae		Leaves	HER: anti-callus (crushed leaves topically applied)		
*Silene vulgaris* (Moench) Garcke, Caryophyllaceae, UNISGSANG15044	Ciuchinot, Cöiet	Leaves		FOO: soup	Fairly common
*Silybum marianum* (L.) Gaertn., Asteraceae, UNISGSANG15045	Cardun	Leaves		HER: hepato-protector (decoction)	Rare
*Solanum dulcamara* L., Solanaceae	Dulcamara	Fruits		HER: bechic (decoction with apples)	Rare
*Solanum nigrum* L., Solanaceae, UNISGSANG15046	Erba morela	Leaves and fruits	HER: anti-arthritis and anti-rheumatic (poultice)	HER: anti-carbuncles (fresh leaves directly applied)	Rare
*Stellaria media* (L.) Vill., Caryophyllaceae	Purota	Aerial parts		FOO: salads	Rare
*Suillus granulatus* (L.) Rousell, Suillaceae	Pinaiolo	Fruiting body		FOO: cooked	Rare
*Tanacetum vulgare* L., Asteraceae	T’nea	Leaves	HER: hypotensive (tea)	VET: digestive and antidote against *Veratrum* spp. ingestion (cows)	Rare
*Taraxacum officinale* (L.) Weber ex F.H. Wigg, Asteraceae, UNISSANG15048	Cicoria de pra, Girasui, Taracun	Leaves and flowers		FOO: salads, omelettes, soups;HER: diuretic, liver protector (tea); bechic (flower syrup)	Very common
*Thymus serpyllum* L., Lamiaceae,UNISGSANG15049, UNISGSANG18032	Serpolet, Serpul	Leaves and flowers	HER: anti-stomatitis (mouthwash)	FOO: meat, soups, and mushroom salads seasoning; smoked with tobacco; FOO/HER: aromatized wines (considered digestive); HER: digestive and tonic (tea); flu (external compress); oral antiseptic (mouthwash of decoction); FOO/HER: liqueurs (considered digestive); VET: fodder for rabbits before they are butchered (for enhancing flavour)	Fairly common
*Tilia cordata* Mill., Malvaceae, UNISGSANG15050	Tij	Leaves and flowers (*tiöl*)	HER: bechic and febrifuge (tea)	HER: bechic (tea)	Very common
*Tragopogon pratensis* L., Asteraceae, UNISGSANG18051	Barbaboch	Leaves	HER: depurative (decoction)	FOO: boiled, soup	Fairly common
*Tussilago farfara* L., Asteraceae	Pata d’asu	Leaves	HER: whooping cough (fumigations); for treating insect bites (compress of the fresh leaves)		
*Urtica dioica* L., Urticaceae, UNISGSANG18055	Urtia, Ürtia	Leaves and roots	HER: anti-dysmenorrhea (decoction of the roots)	FOO: soups, risotto, omelettes, salads (leaves); HER: cicatrizing, anti-bruises (external applications of chopped leaves); anti-alopecia and hair strengthening (decoction, externally applied); VER: enhancing egg production (fodder for hens)	Very common
*Vaccinium myrtillus* L., Ericaceae, UNISGSANG15057, UNISSANG18017	Ambrune	Fruits		FOO: jams; FOO/HER: snack (“good for the eyes”), liqueurs (considered anti-diarrhoeic); HER: anti-prostatitis, antiphlogistic of the urinary tract	Very common
*Vaccinium vitis*-*idaea* L., Ericaceae, UNISGSANG18018	Anghertin	Fruits		FOO: snack	Rare
*Valerianella locusta* (L.) Laterr., Valerianaceae,UNISGSANG15058, UNISGSANG18055	Saladet, Saladet dle funtene	Young leaves		FOO: salads	Very common
*Verbascum thapsus* L., Scrophulariaceae	Verbasco	Leaves and flowers	HER: bechic (infusion of the flowers); anti-arthritis (leaves, externally applied)		
*Veronica allionii* Vill., Plantaginaceae	Te d’montagna	Leaves	HER: tonic (tea)		
*Veronica chamaedrys* L. and *V*. *officinalis* L., Plantaginaceae		Fiori	HER: febrifuge (tea)		
*Viola calcarata* L., Violaceae	Viola, Viuletta d’muntagna	Flowers	HER: bechic (tea)	HER: bechic, laxative, intestinal anti-inflammatory (tea)	Fairly common
*Viola canina* subsp. *montana* (L.) Hartm., Violaceae, UNISGSANG18057	Viulëtta	Flowers		FOO: soup (“müshe morte” = “dead flies”, as the cooked violets resemble flies)	Rare
*Viola odorata* L., Violaceae, UNISGSANG15059	Viola mammola	Flowers		FOO: soups	Rare
*Viola tricolor* L., Violaceae	Viola d’l pensé	Flowers	HER: febrifuge, tranquillizer (tea)		
*Zea mays* L., Poaceae		Stigma	HER: diuretic and antiphlogistic against urinary tract infections (tea);		

FOO recorded wild food plant use, FOO/HER recorded food-medicinal use, HER recorded herbal use for humans, VET recorded veterinary use

Wild food plants and mushrooms still represent an important source of ingredients in the study area today (Table 1). The most important groups of wild food plants are leafy vegetables and mushrooms, which are normally gathered in the spring and autumn, respectively. Most of the recorded wild food and herbal plant reports were previously reported in a few sporadic ethnobotanical studies conducted on the Italian side of the Alps ([60], and references therein [61–65];). Among the traditional and most distinctive recipes, it is worth mentioning the resilience of a soup made with violets called *müshe morte* (“dead flies”), as the cooked flowers resemble flies.

### Cross-temporal comparison

**Fig. 2:**
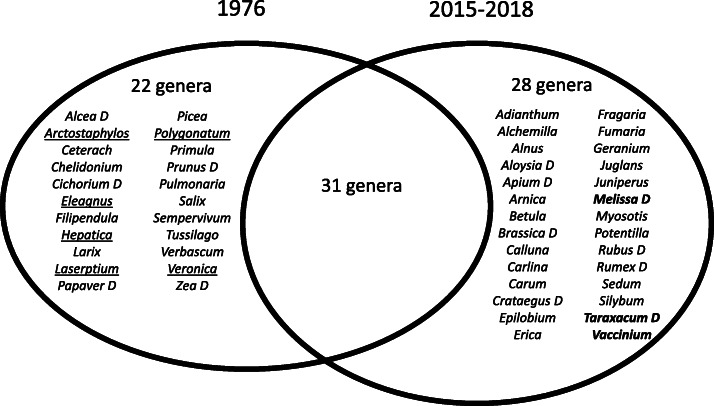
Venn diagram showing the overlap between the medicinal plant genera gathered in the Seventies and those gathered in 2015–2018 in the Sangone Valley. D: cultivated genera or genera growing in anthropogenic environments; genera mainly growing in forest and higher mountain areas are underlined, while genera very commonly used nowadays are in bold type

Figure 2 shows the comparison between the past and current herbal uses of the recorded plant *genera*. We deemed that considering plant genera and not species was more appropriate given the possible discrepancies in species identification between the datasets and taking into account that *under*-*differentiation* does occur for herbaceous plants in the study area (one folk plant name may sometimes correspond to diverse used botanical species within the same genus, see Table 1).

Despite the possible limitations due to the lack of data on the frequency of gathering/use of medicinal plants recorded in the Seventies and the fact that a number of medicinal taxa are not commonly gathered and used nowadays (see Table 1), there was no remarkable quantitative difference between the two medical ethnobotanies (53 recorded herbal genera in the Seventies and 59 in the current study) but qualitatively the difference was substantial.

## Discussion

### The renegotiation of *situated* LEK

Overall, the data analysis suggests a profound change of herbal LEK which is linked to the very way people lived, and now live, in the valley.

The data collected in 1976 show botanical knowledge linked to different ecosystems in the valley, as well as knowledge of specific, local species. Wild species traditionally gathered in more remote mountain areas could be linked to the disappearance of pastoralist and forest-centred activities, which were prominent in the study area many decades ago.

Figure 2 clearly shows that the gathering and use of plant genera that, accordingly to the Italian botanical reference flora [38], mainly grow in pastures, forests and higher mountain areas (underlined in the figure) has ceased, while the collection of *cultivated* medicinal plants or medicinal plants growing in more anthropogenic environments [38] has been newly introduced. This is similar to what has been observed for the patterns of change in wild food plant knowledge in Europe [22, 30].

Thus, the research shows a form of LEK that encompasses the entire complexity of the valley, which is compatible with the specificities of local multifunctional farming. This adaptive strategy [73] involves horticultural activities carried out in fields in the lower parts of the valley, hunting and gathering practiced in the forests and summer dairy farming and milk processing conducted in the high pastures. Moreover, plant knowledge was remarkably focused on medicinal uses of herbs. In a country in which a National Health System was instituted only in 1978 [74], the availability of medical treatments and drugs was limited. In this respect, the use of local plants and remedies was a fundamental resource for families; a resource embedded in and specific to the place.

Forty years later, herbal LEK has been transformed, as has the way the community inhabits the Sangone Valley. Dwelling is the result of the different activities a community or an individual performs in the landscape and on the basis of this activity they experience and know their surroundings thereby developing their knowledge [75, 76]. In this respect, the transformation of LEK is the result of a nonlinear history [77] of demography, education and economy.

On the demographic side, while most of the Alpine Valley in the country faced rapid socioeconomic transformation after WWII [52], which led to the abandonment of the valley [78], the Sangone Valley experienced the opposite. Thanks to its position close to Turin and on the main corridor between Italy and France, like neighbouring Val di Susa [47], during the past 40 years, the valley has been repopulated (see Table 2). While from the 1940s to 1960s local inhabitants abandoned the valley, the repopulation of the valley is linked first of all to the opening of a large car manufacturing facility in Rivalta in 1967, as well as other factories in other municipalities in the Sangone Valley [79]. Due to this industrialization, new factory workers, coming from all across Italy [80], have settled in the municipalities of the valley since the 1960s. Moreover, since the 1980s, the valley has been the destination of a new wave of immigrants. In this case, it has been mostly professionals and other people from Turin, who decided to move away from the city looking for better accommodation and environment. Due to the landscape and overall affordability of houses, these newcomers settled in the valley, enriching the cultural diversity of the place. In regard to LEK, these new inhabitants, most of the time, have had little or no knowledge of the valley and its environment but have enriched the culture of the place by bringing with them knowledge concerning different plants from their home communities [81].

**Table 2:** Population change in the municipalities of the Sangone Valley

Municipality	Inhabitants			Population change in %		
	1951	1971	2011	1951➔2011	1951➔1971	1971➔2011
Coazze	3480	2819	3084	− 11.38	− 18.99	9.40
Valgioie	631	311	948	50.24	− 50.71	204.82
Giaveno	8835	10641	16281	84.28	20.44	53.00
Trana	1610	1792	3881	141.06	11.30	116.57
Reano	808	832	1689	109.03	2.97	103.00
Sangano	549	1367	3807	593.44	149.00	178.49
Bruino	786	3362	8479	978.75	327.74	152.20
Rivalta	2174	10358	19245	785.23	376.45	85.80
TOTAL	18873	31482	57414	204.21	66.81	82.37

From ISTAT/National Italian Institute of Statistics data collected during the 1951, 1971 and 2011 national census surveys

This period has not only transformed the demography of the place but also deeply affected the family economy of the valley. As in other parts of the Western Alps [82], it has coincided with a deep transformation of the local economy. In particular, it is linked to a crisis of the multifunctional farming system. Attracted by new and more lucrative jobs in industry or the service sector, locals have abandoned agriculture as the main field of activity. At the same time, the agricultural sector has moved towards industrialization, abandoning traditional practices or marginalizing their role. The same has happened with the role of wild plant and mushroom gathering, which have been relegated to being a marginal hobby with the sole main exception of mushroom harvesting, and in particular boletus mushrooms, which have become a key landmark product for the Sangone Valley, celebrated through advertising and local events, such as the mushroom food festival in Giaveno.

Finally, on the cultural side, as in the rest of the region, the second half of the twentieth century marked a crisis in the transmission of traditional knowledge, as well as environmental knowledge [83]. The crisis was not linked just to the passage from orality to written knowledge, a process that has been vastly explored [11, 84–86], but rather to the expansion and differentiation of (written and oral) sources that compose the mediascape [87] of a local community. In this regard, current environmental knowledge is not the result of a linear intergenerational transmission of knowledge, nor just the result of a process of legitimate peripheral legitimation undergone by the individual within the local community [88]. Rather, it is the assemblage of information gathered by individuals based on their interaction with other members of the community as well as with experts, their formal education and popular literature and also with the information gathered from mass media.

Moreover, “global” remedies emerged as a new phenomenon with three genera (*Melissa*, *Taraxacum*, *Vaccinium*; highlighted in bold type in Fig. 2) that are now widely used but were not even collected forty years ago. In particular, the gathering and use of *Alchemilla*, *Crataegus*, *Melissa*, *Silybum*, *Aloysia*, *Betula*, *Fumaria*, *Taraxacum* and *Vaccinium* spp. may have been spread via popular herbal literature printed during the past four decades in Italy [66–69, 89], possibly reinforced first by TV programs and later, during the past decade, by some popular Internet sources and social media [70–72]. In the case of *Epilobium angustifolium*, a research group has, for example, recently linked the increase in popularity of its herbal use in Eastern European countries to a clear influence of popular literature, likely spread by the work of Austrian herbalist Maria Treben, whose writings have been best-sellers in many countries during the past three decades, and also by a number of Russian internet sources [90].

Undoubtedly, the changes that the valley has experienced have transformed local communities and their knowledge. In light of this change, the transformation in LEK this research has pointed out does not suggest a *de*-*situated* local environmental knowledge, i.e. depleted and eroded by modernity, as commonly assumed in the literature after the postmodernist turn triggered by the seminal work of Lyotard [91]. Our research, instead, suggests that current herbal LEK is the result of a different way of living in the surroundings that answers to present-day social, economic and cultural needs. As pointed out by Nygren [92], LEK is not antithetical of globalization and modernity, but rather it is embedded in this complexity. The making of this knowledge is complex and comes full of friction [93], through which experiences and knowledge developing inside and outside the locale are assembled. Moreover, the observed co-evolution of LEK reflects the transformation of the local socio-ecological system [7]. Thus, the case of the Sangone Valley shows the plasticity and dynamicity of LEK and outlines a circular relationship between environmental change, social change and LEK in which LEK is mutating in response to the transformations of society and the environment. On the other hand, their change also modifies the very way in which the community sees its society and its surroundings.

Despite the major limitation of our study (the lack of data concerning the frequency of gathering/use of the medicinal plants 40 years ago), this circularity suggests that herbal LEK in the study area is not simply eroded or disappearing, but tangible and present, and thus always, but differently, *situated*. These findings challenge, to some extent, the main conclusions of most ethnobotanical studies that demonstrate or postulate an erosion of medicinal plant knowledge everywhere in the world, especially among the younger generations. This assumption should perhaps be analysed with more caution, also because nature-related knowledge may be affected by the so-called *shifting baseline syndrome* [94]; that is, fluctuations in several factors (such as the availability of plant resources and plant knowledge that can differ over the course of a lifetime) may cause changes in the reference (baseline) of different generations [94].

Moreover, this finding seems to confirm what Vandebroek and Balick [95] pointed out a few years ago in their pioneering work on the botanical knowledge of Dominican migrants in NYC, in which they showed a statistically measurable increase of knowledge associated with migration and urbanization. The findings are in agreement also with those showing the Estonian evolution of herbal plant knowledge during the past century, during which the use of medicinal plants depending on human influence was remarkably amplified [23].

Ultimately, our data suggest that future directions of research should more carefully look at the *adaptive capacity of LEK systems* [20], especially in urban environments or sites influenced by urban processes, and avoid the assumptions that some folklore-driven approaches have too hastily proposed by romanticizing “the good old times”.

### LEK and rural development in the Alps: from museums to participatory planning?

If the above is true, however, what is the role of LEK in alpine development and the development of the study valley?

In the Sangone Valley, as well as in other areas of the Western Alps, LEK was mostly interpreted as a fragment of intangible heritage; pieces of ancient knowledge preserved only in fragments [96–99]. With this approach, LEK has been placed at the centre of a process of *heritagisation* and museification, culminating in the development of ethnographic museums as well as specific tourism and commercial communication campaigns [56, 100]. In this sense, LEK has been turned into an ethno-commodity [101] aimed at addressing cultural and economic issues related to tourism and food business development. However, as pointed out by Comaroff and Comoraff [102], such an approach, even if rewarding in the short term, furthers the cultural and economic dependence of the local community on the outside because it triggers a vicious circle of cultural production in which local communities promote and inflate traditional knowledge in order to suit the needs of tourists and the market. Thus, despite the lexicon and premise, this approach to LEK risks not fostering a real opportunity for sustainable development.

Moving away from this approach by recognizing the importance of LEK as an expression of the actual and current relationship between community and environment, LEK becomes an essential tool for the interpretation and planning of the alpine landscape. In a context in which dynamic, decentralised, participatory planning is recognised as a key tool for sustainable development [102], LEK and its diachronic analysis become a tool for interpreting the transformation a region is undergoing. In particular, LEK can contribute to understanding the movement of the anthropogenic frontier, and therefore the abandonment or intensification of the use of areas in the territory. In our research, this is the case, for example, for the forest and the higher areas of the Sangone Valley. Local ecological knowledge can also help to interpret transformation in the use of the landscape that is not linked directly to economic dynamics, but rather to cultural change. In our research, this is the case for the impact of new popular literature and media in shaping local botanical knowledge, expanding proficiency with exotic plants in front of the loss of knowledge of native species. Lastly, LEK can contribute to developing targeted initiatives in environmental conservation and rural development programs.

Overall, this approach supports the design of better initiatives in the field of rural development, fully embracing the challenge presented by the social change the entire Alps is facing.

## Conclusions

This paper has focused on the transformation of LEK in the Sangone Valley, using folk medico-botanical knowledge as a proxy. The analysis pointed out that the change that botanical knowledge and practices concerning medicinal plants have undergone are mainly represented by ceasing collection and use of a few plants growing in higher mountain environments and by the introduction of new customs linked to species growing in anthropogenic milieus or spread by popular herbal sources. These changes are possibly linked to the social, cultural and economic transformations the communities of the valley have faced over the past 40 years. In light of this complex interconnection, the presented data suggest that LEK has not been eroded, but re-arranged. Moreover, these changes, even though profound, have not *de*-*situated* LEK from the valley, but rather have defined a new configuration in the relationship between communities and their environmental surroundings. In this respect, we argue that the present configuration of LEK should not be considered a corrupted or fragmentary form of a romanticized ancient and pristine condition. The complex connections between changes in LEK systems and the social, cultural and economic transformations that the valley experienced in the past few decades further suggest that the value of local botanical knowledge does not merely lie in its heritagisation, but rather in its germane implementation as a tool for fostering resilient strategies of sustainable development.

## Data Availability

Original dataset of the work published in 1977 is available at the authors’ address and in Table 1. Datasets of the work conducted in 2015 and 2018 are presented in Table 1.
